# Acute cocoa Flavanols intake has minimal effects on exercise-induced oxidative stress and nitric oxide production in healthy cyclists: a randomized controlled trial

**DOI:** 10.1186/s12970-017-0186-7

**Published:** 2017-08-10

**Authors:** Lieselot Decroix, Cajsa Tonoli, Danusa Dias Soares, Amandine Descat, Marie-José Drittij-Reijnders, Antje R. Weseler, Aalt Bast, Wilhelm Stahl, Elsa Heyman, Romain Meeusen

**Affiliations:** 1 Human Physiology Research Group, Faculty of Physical Education and Physical Therapy, Vrije Universiteit Brussel, Brussels, Belgium; 2 Department of Physical Activity, Muscle and Health, Faculty of Sport Sciences and Physical Education, Université de Lille, Lille, France; 3 Department Rehabilitation sciences and kinesitherapy, Faculty of Physical Education and Physical Therapy, Universiteit Gent, Ghent, Belgium; 4 Department of Physical Education, University of Minas Gerais, Belo Horizonte, Brazil; 5 Center of measurements and analysis (CMA), Facultyof Pharmaceutical Sciences, Université de Lille, Lille, France; 6 Department of Pharmacology and Toxicology, Maastricht University, Maastricht, the Netherlands; 7 Institute of Biochemistry and Molecular Biology I, Faculty of Medicine, Heinrich-Heine University Dusseldorf, Dusseldorf, Germany

**Keywords:** Cocoa, Flavanols, Oxidative stress, Nitric oxide, Exercise

## Abstract

**Background:**

Cocoa flavanols (CF) can stimulate vasodilation by improved nitric oxide (NO) synthesis and have antioxidant and anti-inflammatory capacities. This study aimed to examine whether acute CF intake can affect exercise-induced changes in antioxidant capacity, oxidative stress, inflammation and NO production, as well as exercise performance and recovery in well-trained cyclists*.*

**Methods:**

Twelve well-trained male cyclists (mean ± SD age, VO2max: 30 ± 3 years, 63.0 ± 3.5 ml/kg/min) participated in this randomized, double-blind, cross over study. On 2 separate occasions, subjects performed two 30-min time trials 1.5 (TT1) and 3 (TT2) hours after CF (900 mg CF) or placebo (PL, 13 mg CF) intake, interposed by passive rest. Lactate, glucose, heartrate, rating of perceived exertion (RPE) and power output were measured during the TTs. Blood was drawn at baseline, before and after each TT and analyzed for epicatechin serum concentrations, trolox equivalent antioxidative capacity (TEAC), uric acid (UA), malonaldehyde (MDA), L-arginine/ADMA, citrulline, interleukin (IL)-1, IL-6 and tumor necrosis factor (TNF)-α plasma concentrations. Relative changes in blood markers and pacing strategy during TT were analysed by repeated measured ANOVA. TT performance was compared between PL and CF by paired t-test.

**Results:**

Epicatechin concentrations were increased by CF intake. Exercise-induced increase in TEAC/UA was improved by CF intake (F(1) = 5.57; *p* = .038) (post-TT1: PL: 113.34 ± 3.9%, CF: 117.64 ± 3.96%, post-TT2: PL: 108.59 ± 3.95%, CF: 123.72 ± 7.4% to baseline), while exercise-induced increases in MDA, IL-1 and IL-6 were not affected by CF intake. TNF-α was unaltered by exercise and by CF. Exercise-induced decreases in L-arginine/ADMA and increases in citrulline were not affected by CF intake. TT1 and TT2 performance and exercise-induced physiological changes were unaffected by CF intake.

**Conclusion:**

Acute CF intake increased total antioxidant capacity in rest and during exercise, but did not affect exercise-induced lipid peroxidation, inflammation, nor NO production in healthy athletes. Acute CF intake did not improve TT performance and recovery.

**Trial registration:**

ISRCTN32875, 21-11-2016, retrospectively registered.

## Background

A balanced and well-chosen nutrient intake is not only crucial for a healthy lifestyle, but also for optimal sport performance. There is a widespread use of dietary antioxidants, including vitamin E, resveratrol, beetroot, quercetin and cocoa flavanols (CF) in the athletic field. The main flavanols in cocoa are epicatechin, catechin (monomers) and procyanidins (oligomers). Their antioxidant (and in some situations perhaps pro-oxidant) effect is determined by the tricyclic structure of the flavanols. In vitro and in vivo studies clearly show that CF have a strong antioxidant capacity [1, 2]. However, the potential role of chronic or acute CF intake to counteract exercise-induced oxidative stress and its effect on exercise performance is still unclear [3–7]. Oxidative stress refers to the imbalance between oxidants and antioxidants in favor of the oxidants, leading to a disruption of redox signaling and/or molecular damage [8]. During exhaustive exercise, NADPH oxidase-derived formation of reactive oxygen species (ROS) results in an altered redox state in the muscle [9]. In contrast to the initial belief that nutritional antioxidant intake would be beneficial for exercise performance by protecting the body against oxidative stress, it is now hypothesized that exercise-induced oxidative stress promotes the expression of antioxidant defence and the adaptive responses to training [10]. However, when exercise-induced ROS formation exceeds the antioxidant capacity, oxidative stress occurs with detrimental effects on proteins, lipids and DNA, possibly leading to contractile muscle dysfunction, accelerated muscle-fatigue and reduced exercise performance [11]. Thus, at moments when optimal exercise performance is key and training adaptations are of minor importance (e.g. competition), acute intake of antioxidants may help scavenge free radicals and therefore directly prevent a decline in exercise performance and optimize post-exercise recovery [9, 12].

In the recent years, CF have also been discovered as potent anti-inflammatory agents [13]. Exhaustive exercise can cause inflammation [14], resulting in a potential role for CF as a nutritional intervention to prevent exercise-induced muscle damage. Nevertheless, Allgrove et al. [6] did not find any beneficial effect of 2 week CF (108 mg) intake on exercise-induced inflammatory markers.

Independent from its antioxidant and anti-inflammatory properties, CF are also known to stimulate vasodilation through increased nitric oxide (NO) availability [15]. In vitro experiments demonstrated that CF lead to the inhibition of arginase, resulting in a greater L-Arginine availability, the substrate of endothelial NO synthase (eNOS) [16]. In vitro [17] and in vivo [18, 19] experiments showed that CF intake activates eNOS, which converts L-Arginine, in the presence of molecular oxygen, to NO and citrulline. Besides its role in vasodilation, NO can also react at a diffusion-controlled rate with superoxide to form the highly reactive oxidant peroxynitrite. Consequently, by elevating NO production, CF might hypothetically have a pro-oxidant effect. However, because of its antioxidant capacity, CF can reduce ROS formation (e.g. superoxide) and repress the formation of peroxynitrite, thus elevating NO availability [20]. Based on the fact that NO modulates blood flow and mitochondrial respiration during exercise [21, 22], it has been suggested that increased NO production may enhance oxygen and nutrient delivery to active muscles, as well as improve mitochondrial efficiency, hence improving tolerance to physical exercise and recovery mechanisms [21]. Although many athletes use supplements of NO because of their potential ergogenic role, the scientific evidence is very scarce [21]. eNOS activity and thus NO production are stimulated by exercise-induced shear stress and thus strongly depend on training status [23]. This suggests a minimal effect for CF and other nutritional supplements on eNOS activity and NO production in healthy trained athletes without vascular restrictions. Whether CF supplementation can alter NO production during exercise, and whether it may improve exercise performance and recovery in well-trained athletes, remains elusive [5–7].

Therefore, the aims of this study were to examine the effects of CF intake on: *i)* indirect markers of eNOS dependent NO synthesis, L-arginine and citrulline, during exhaustive exercise *ii)* exercise-induced changes in antioxidant capacity, oxidative stress and inflammation, and *iii)* exercise performance and recovery in well-trained athletes. We hypothesized that CF intake *i)* will have little effect on indirect markers of NO production and exercise performance in these healthy, well-trained, subjects, but *ii)* will decrease exercise-induced oxidative stress and inflammation, leading to an improved recovery.

## Methods

### Participants

Twelve well trained male cyclists (Performance level 3 [24]) (mean ± SD age 30 ± 3 years, height 177.9 ± 8.8 cm, body mass 72.8 ± 7.8 kg, and VO_2_max 63.0 ± 3.5 mL/kg/min) participated in this randomized, double blind, cross-over interventional trial. Volunteers were excluded from the study if they met any of the following exclusion criteria: (1) < 20 years or >35 years, (2) hypertension, (3) cardiovascular disease, (4) smokers or history of smoking, (5) habitual antioxidant supplementation. The experimental procedures and potential risks were explained to the participants and a written informed consent was provided and signed prior to inclusion in the study. The study protocol was approved by the research ethical committee of the Vrije Universiteit Brussel and the study was conducted according to the Declaration of Helsinki (1964).

### Study design

Subjects visited the lab 3 times during 3 consecutive weeks, with 7 days in between. Training, food intake and lifestyle were kept constant during those 3 weeks. Subjects were asked to complete a 24 h–food recall on 5 random days during the study period (to avoid a potential influence of regular polyphenol intake on the measurements) and training intensity and duration were registered in a training diary. Participants were asked to record their dietary in- take of the last 24 h before each lab visit and to replicate this diet in the 24 h preceding each test. On the first visit, subjects underwent a complete medical screening and performed a maximal incremental cycle test (initial workload of 80 W, increased every 3 min by 40 W until volitional exhaustion) on an electromagnetically braked cycle ergometer (Lode, Groningen, The Netherlands). Peak power output (PPO) was determined and maximal oxygen uptake (VO_2_max) was measured using the Metalyzer Cortex (Germany).

**Fig. 1:**
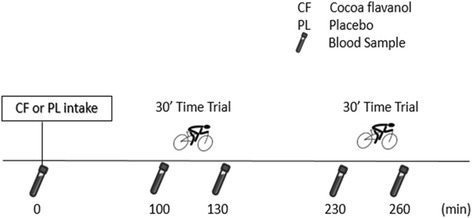
Study protocol of the 2 interventional trials, performed in randomized order with cocoa flavanol (CF) or placebo (PL), with a washout-out period of 1 week in between. During the time trial, subjects had to cover an amount of work (equal to 75% of peak power output during 30 min) as fast as possible

Subsequently, subjects underwent 2 interventional (exercise) trials where they consumed a CF or placebo (PL) drink (300 ml), in a randomised order with 1 week in between. Subjects reported to the lab in a 4 h–fasted state at 12 pm. Subjects were asked not to perform heavy exercise in the last 48 h and to abstain from caffeine and foods with a high polyphenol content (green tea, grapes, olives, dark chocolate, hazel and pecan nuts, berries) for the last 24 h. Baseline blood samples were collected, subjects were weighed and blood pressure was taken. Subjects then consumed either a high CF-content chocolate milk (CF - 903.75 mg CF, Acticoa®) or the placebo low CF chocolate milk (PL – 15 mg CF) which were matched in taste, color, calories, macronutrients, caffeine and theobromine. The exact composition of the CF and PL drink, including monomers (epicatechin, catechin) and oligomeric proanthocyanidins are depicted in Table 1. This drink was consumed together with a standardized lunch, carefully selected by a nutritionist to contain a high amount of carbohydrates to increase CF absorption [25] (80% carbohydrate, 15% protein, 5% fat, 10 kcal/kg bodyweight). As the peak blood concentration of CF is reached 2 h after consumption of the drink [25], a 2nd blood sample was taken 100 min later. Subsequently, a 30-min TT was started. Immediately after the TT, a 3rd blood sample was taken. A 4th sample was taken after a 100-min passive recovery and consequently a 2^nd^30-min TT was started. Immediately after the 2^nd^TT, a 5th blood sample was taken (Fig. 1).

**Table 1:** Nutritional profile of cocoa powder (solved in 300 ml skimmed milk)

	Cocoa flavanol (CF)	Placebo (PL)
Total Flavanols (mg)	900	15
- (−)-epicatechin	185	0
- (+)-catechin	20	0
- Procyanidins dimer	190 (21.9%)	
- Procyanidins trimer	117 (13.0%)	
- Procyanidins tetramer	113 (12.5%)	
- Procyanidins pentamer	71 (7.9%)	
- Procyanidins hexamer	52 (5.8%)	
- Procyanidins heptamer	51 (5.7%)	
- Procyanidins octamer	39 (4.3%)	
- Procyanidins nonomer	33 (3.7%)	
- Procyanidins decamer	25 (2.8%)	
Cocoa powder (g)		
- Acticoa powder	12.0	0.0
- Alkalized cocoa powder	3.0	15.7
- Potassiumchloride (KCl)	0.7	0.0
- Sugar	35.0	35.0
Protein (g)	3.2	2.9
CHO (g)	38.7	38.4
Fat (g)	2.3	2.9
Kcal	193.4	193.4
Caffeine (mg)	30	30
Theobromide (mg)	315	315
Cadmium	<1 ppm	<1 ppm

### Measurements

#### Time trial

After a standardised warm up of 5 min at 120 W, subjects were instructed to cover a fixed amount of work (the equivalent work of 75% of peak power output during 30 min) as fast as possible. The initial workload of the TT corresponded to 75% of the peak power output (280 ± 27.6 W), but subjects were free to change their power output as desired from the outset. If the power output decreased, the duration of the time trial increased. No feedback regarding time lapse, power output, heartrate or pedal cadence was given, except for total workload that was completed. Time to complete the TT and physiological parameters as lactate, heartrate, and rate of perceived exertion (RPE) were used as primary outcome measures to determine the effect of CF on exercise performance (data from TT1) and recovery (data from TT2). Power output and heartrate were constantly registered during the TT. Relative power output (percent of peak power output), was calculated every 5 min of the TT. Thus, changes in relative power output were analysed at 7 time points during each TT. Lactate, glucose and RPE were measured at the start, after 10 and 20 min, at the end of the TT and 5, 10 min after the TT.

#### Blood collection and analyses

A catheter was placed in a antecubital vein upon arrival at the lab. Venous blood samples were collected at baseline, 100 min later (pre-TT1),immediately after TT1 (post-TT1), 100 min after finishing TT1 (pre-TT2) and immediately after TT2 (post-TT2). Blood was collected in two 5 ml EDTA tubes, one 5 ml heparinized tube and one 8 ml anticoagulant-free tube and centrifuged immediately to obtain plasma or after 30 min at room temperature to allow clotting to obtain serum (10 min at 3000 rpm, 4 °C). Plasma and serum were aliquoted and stored at −80 °C until further analyses. All biochemical data were corrected for changes in plasma volume using the determination of haematocrit and the concentration of haemoglobin according to Dill and Costill [26]. Pre-and post TT1 and pre-and post TT2 values were normalized to the baseline values and expressed as % change from baseline. Blood lactate and glucose were measured in a capillary earlobe sample. Lactate was analysed by a Biosen 5030 (EKF, Magdeburg, Germany) and glucose by a photometrical method (using the hexokinase Roche).

##### Determination of serum flavanols concentration

Serum samples were analysed for epicatechin and catechin concentrations as described by Warden et al. [27]. In detail, 0.5 ml of serum was mixed with 1.0 ml phosphate buffer (100 mM, pH 5, containing ascorbic acid (20 mg/ml), EDTA (1.5 mg/ml)) and 20 μl glucuronidase/sulfatase (100,000 and 7500 units/ml, respectively) and incubated at 37 °C for 30 min to hydrolyse glucuronate and sulfate conjugates of epicatechin and catechin. Then, 5 ml tert-butylmethylether was added and vortexed for 1 min. For phase separation, the mixture was centrifuged at 10 °C for 5 min at 5000 rpm.

The organic phase was transferred to a new tube and after a second extraction with 5 ml tert-butylmethylether, the combined extracts were dried under a stream of nitrogen and stored at −80 °C. For HPLC analyses, the dry residues were reconstituted in 200 μl methanol, vortex-mixed and centrifuged at 5 °C at 14.000 rpm for 5 min. 20 μl of the supernatant were injected onto the HPLC-column. For HPLC analysis, a reversed-phase RP18 end-capped column (Lichrospher 100, 5 μm, 250 × 4 mm; Merck) coupled with a guard column (Lichrospher 100, 5 μm, 4 × 4 mm; Merck) was used. Detection was accomplished at an excitation wavelength of 280 nm and an emission wavelength of 310 nm. Data were recorded by HPLC-System Manager software (Merck/Hitachi; Darmstadt, Germany). Samples were eluted from the column at 20 °C using a step-gradient as follows: From 0 to 18 min 53% acetonitrile/H_2_O/acetic acid (150:846:4)/47%H_2_O/acetic acid (1000:5), from 18 to 27 min 80% acetonitrile/H_2_O/acetic acid (150:846:4/20% H_2_O/acetic acid (1000:5). To elute retained compounds the column was flushed from 27 to 60 min with 100% acetonitrile/acetic acid (1000:4) and subsequent equilibration from 60 to 80 min with starting conditions. Flow rate was 1.5 ml/min. Under these conditions the analytes elute with retention times of 15.4 min for catechin and 24.9 min for epicatechin. Peak areas of catechin and epicatechin were used to calculate the concentrations applying the external standard method. Standard curve linearity was observed in the range from 0.125 to 20 μM for both compounds.

##### Quantification of total antioxidant capacity (TEAC), uric acid (UA) and lipid peroxidation (MDA) in plasma

Plasma antioxidant capacity was quantified as trolox equivalent antioxidant capacity (TEAC) according to Fischer et al. [28] and corrected for plasma uric acid (UA) as a major antioxidant in the blood [29]. In this procedure, the decolorization of the preformed green-blue 2,2`-azino-bis (3-ethylbenzthiazoline-6-sulfonic acid) radical (ABTS^•+^) by the sample within a fixed amount of time reflects the antioxidant capacity of the blood sample.

##### Quantification of inflammatory markers in plasma

The inflammatory cytokines tumor necrosis factor (TNF)-α, interleukin (IL)-1 and IL-6 were quantified by means of commercially available ELISA kits (PeliKine Compact human ELISA kits, CLB/Sanquin). The limits of sensitivity were 4 pg/ml for TNF-α, 2.5 pg/ml for IL-1 and 0.5 pg/ml for IL-6.

##### Determination of mediators of NO-pathway (citrulline, L-arginine, ADMA)

An internal standard (150 μL, 1 μM ADMA and 50 μM Arginine, methanol mixture) was added to 10 μL of plasma and centrifuged (13,000 rpm, 10 min, 4 °C) to remove the precipitated proteins. Supernatant was collected and dried under a stream of nitrogen at 70 °C. The dried extract was dissolved in 100 μL of a butanol solution containing 3 N HCl and kept at 70 °C for 40 min. The solvent was removed by evaporation under nitrogen flow at 70 °C. The sample was then dissolved in 2.5 mL of water-methanol (90:10, v:v) containing 0.1% formic acid and 5 μL was injected into an analytical column (Kinetex C18 (5 μm, 2.1x100mm)).Mass spectrometric analysis was performed using an UFLC-XR Shimadzu coupled with an QTRAP® 5500 hybrid system, equipped with a Turbo VTM ion197 source (AB Sciex, Foster City, CA, USA). Multiple reaction monitoring (MRM) measurement was performed using optimal cone and collision energy values. The run was performed at a flow rate of 500 μL*min^−1^at 30 °C, lasting 9 min in total. A gradient profile consisted of solution A (water with 0.1% (*v*/v) formic acid) and solution B (methanol with 0.1% (*v*/v) formic acid). The percentage of organic solution B was changed linearly as follows: 0 min, 2%; 4 min, 7%; 6 min, 50%; 7 min, 2%; 9 min, 2%. Data were acquired with Analyst Software version 1.5.2. Calibration curves, performed in water, were obtained adding increasing concentrations of ADMA and SDMA from 0.1 to 4 μM and L-arginine and citrulline from 12.5 to 125 μM.

### Sample size calculation and statistical analysis

The minimal sample size was calculated using G*Power using results from previous studies examining effects of CF intake on markers of oxidative stress and exercise performance [3, 5, 7]. To detect a difference at a power of 0.8 with 95% confidence, a minimal sample size of 12 individuals with each intervention was necessary. Statistical analysis was performed with IBM SPSS Statistics (version 22; IBM Corp., Armonk, N.Y., USA) and considered significant at α = 0.05. Normality of the data was tested with the Kolmogorov-Smirnov test. Data are presented as mean ± standard deviation. A paired samples t-test was used to determine differences in TT performance. Since relative power output during the time trial was not normally distributed, non-parametric Friedman and Wilcoxon signed rank tests were used to determine differences in pacing strategy between CF and PL intake. To correct for the large inter-individual variation in some plasma markers, relative changes (% change to baseline) were calculated and used for data analysis. To examine the effects of CF and exercise on physiological markers, plasma flavanol metabolites and markers of oxidative stress and inflammation, two-way repeated measures (trial x time) analysis of variance (RM-ANOVA) was performed. To evaluate the effect of CF intake on markers of the NO-pathway before and after exercise, 2 (trial)*3 (time; baseline, before and after TT1) RM-ANOVA was used.

## Results

### Epicatechin/catechin serum concentrations

**Fig. 2:**
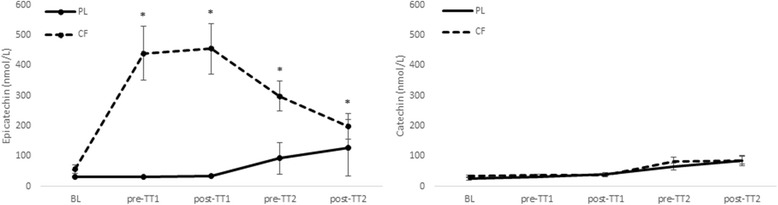
Serum epicatechin and catechin concentrations (expressed in nmol/L) after cocoa flavanol (CF) or placebo (PL) intake, before and after the two time trials (TT). *: *p* < 0.05 compared to baseline (BL)

CF intake significantly increased serum epicatechin concentrations before and after TT1 (100 min and 130 min after intake), as well as before and after TT2 (230 and 260 min after intake), compared to baseline (main effect of time: F = 13.23, *p* < 0.001). The peak concentration was reached before and after TT1. After PL intake, epicatechin levels remained unchanged (Fig. 2). Serum catechin concentrations did not significantly differ between CF and PL intake and between the different time points.

### Plasma concentrations of mediators of the NO-pathway

**Fig. 3:**
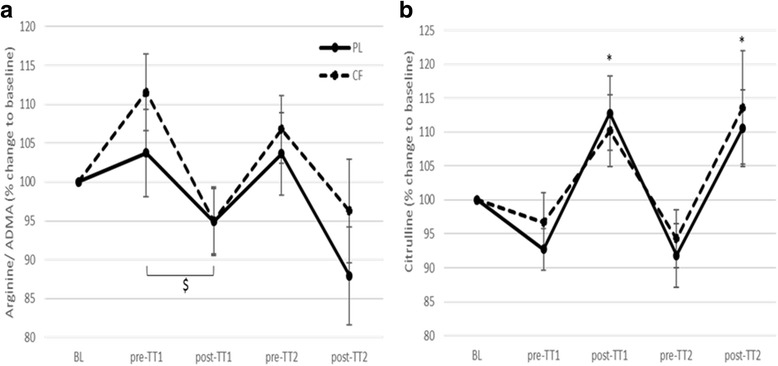
Influence of cocoa flavanols (CF) and exercise on plasma concentrations of arginine/ADMA and citrulline, mediators of the NO-pathway. (**a**) Both time trials (TT1 and TT2) decreased arginine/ADMA concentrations after CF and placebo (PL) intake. The decrease in arginine/ADMA concentrations, induced by TT1, was significantly larger after CF intake, compared to PL intake. (**b**) Citrulline concentrations were significantly increased after TT1 and TT2 in the CF and PL trial. $: *p* < 0.05 for interaction effect in 3*2 RM-ANOVA. *: *p* < 0.05 compared to the previous time point. Mean ± SEM presented

A main effect of time was found for L-arginine/AMDA and citrulline plasma concentrations (RM-ANOVA (5*2), L-arginine/ADMA: F(4) = 3.69; *p* = .01; citrulline: F(4) = 11.88; *p* < .001)). However, no effect of trial nor an interaction effect were observed. Post-hoc analysis showed that exercise (both TTs) significantly decreased L-arginine/ADMA and increased citrulline concentrations in plasma (Fig. 3).

### Markers of oxidative stress

Plasma concentration of UA was significantly increased by CF, compared to PL, as indicated by a significant main effect of trial (F(1) = 7.21; *p* = 0.02). UA was increased after TT1 and TT2 compared to before TT1 and TT2 and was significantly lower before TT2, compared to after TT1 (main effect of time (F(4) = 17.7; *p* < 0.001)) (no interaction effect).

Exercise and CF intake elevated total plasma antioxidant capacity reflected by the TEAC values corrected for UA concentrations (TEAC/UA) (main effect of time (F(4) = 11.84; *p* < 0.001), main effect of trial (F(1) = 5.57; *p* = .038) (no interaction effect). Post-hoc analysis showed that TEAC/UA was significantly higher in the CF trial than in the PL trial. After each TT, TEAC/UA was significantly increased compared to the concentrations before the TT. Before TT2, TEAC/UA was significantly lower compared to after TT1.

**Fig. 4:**
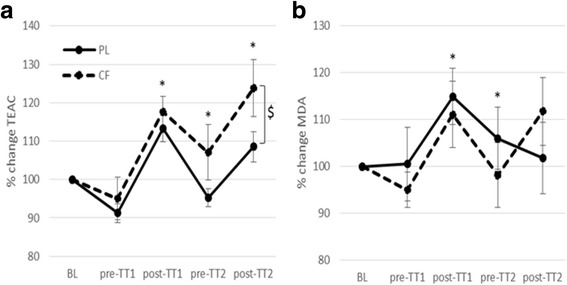
Relative changes in total antioxidant capacity of plasma (TEAC/UA) (**a**) and plasma malondialdehyde concentrations (MDA) (**b**) in response to cocoa flavanols (CF) or placebo (PL) and 2 time trials (TT). $: *p* < 0.05 CF vs PL. *: *p* < 0.05 compared to the previous time point. Mean ± SEM presented

MDA plasma concentration significantly changed over time, but was not affected by CF intake (effect of time: (F(4) = 2.98; *p* = 0.029), no effect of trial/interaction effect). Post-hoc analysis showed that MDA concentrations were significantly higher after TT1 compared to before TT1 and compared to before TT2 (Fig. 4).

### Plasma markers of inflammation

Neither CF intake, nor exercise changed TNF-α (data not shown). For IL-1 and IL-6, a main effect of time was observed (IL1: F(4) = 2.96; *p* = .03; IL6: F(4) = 18.88; *p* < 0.001). Post-hoc analysis revealed that IL-1 and IL-6 were both increased after TT1 (IL-1, PL:+ 12.2 ± 16.0%; CF: + 12.8 ± 12.6%) (IL-6, PL:+ 64.9 ± 10.5%; CF: + 70.2 ± 14.2%). However, TT2 did not induce any significant increases in IL-1 or IL-6 (data not shown). CF intake did not influence IL-1 or IL-6 (no main effect of trial, no interaction effect).

### Impact of CF intake on exercise performance: Performance and pacing strategy during TT1

**Fig. 5:**
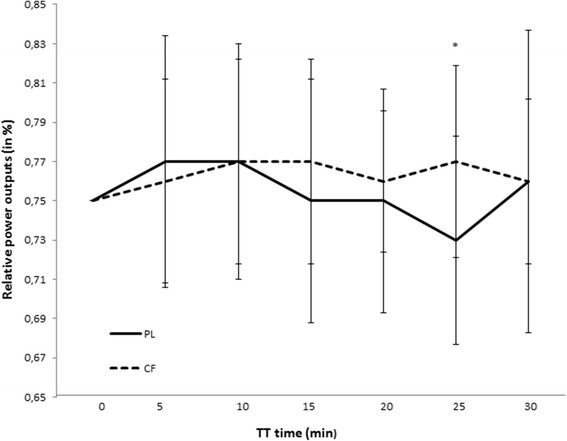
Time trial performance. Pacing strategy of TT1 after cocoa flavanol (CF) or placebo (PL) intake. The relative power output after 25 min was significantly higher after CF intake compared to PL intake. *: *p* < 0.05 compared to PL

Time to complete TT1 tended to be faster after CF intake compared to PL intake, although not significant (PL: 29′47″ ± 1′58″; CF: 29′13″ ± 1′19″; *p* = 0.09). The difference in mean power output during TT1 was +3 ± 8 W (+1.22 ± 3.03%) after CF intake compared to PL. This increase is smaller than the typical error of measurement for a TT and thus not relevant to improve performance in real-life. Friedman test showed no significant changes of pacing strategy at 7 different time points in the PL trial and in the CF trial. Wilcoxon signed rank test showed no significant differences in relative power outputs between CF and PL at the start, 5, 10, 15, 20 min and the end of the TT. A significant higher power output in the CF trial, compared to the PL trial, was found after 25 min in TT1 (PL: 73.09 ± 5.3%;CF: 76.75 ± 4.9%; *p* = 0.03; absolute difference: +14 ± 0.3 W after CF intake (280 ± 29 W) compared to PL (266 ± 29 W) (Fig. 5).

**Fig. 6:**
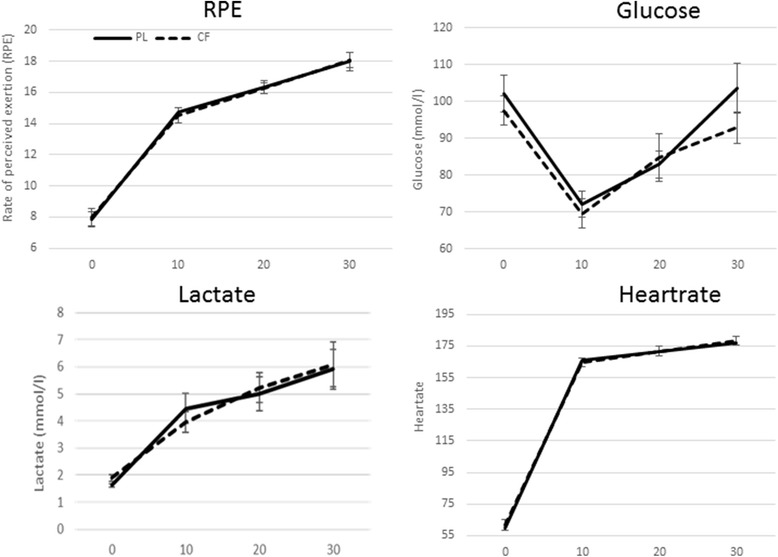
Physiological parameters during time trial 1 (TT1). No significant differences in rate of perceived exertion (*RPE*), *lactate*, *glucose* or *heartrate* during TT1 were seen after cocoa flavanol (CF) compared to placebo (PL) intake

During TT1, RM ANOVA (time (5)*trial (2)) for blood lactate, glucose and RPE showed a main effect of time (*p* < 0.001), but no significant main effect of trial and no significant interaction effect. RM ANOVA (time (4)*trial (2)) for heartrate showed a main effect of time (F = 841.38; *p* < 0.001), but neither significant main effect of trial nor significant interaction effect (Fig. 6).

### Impact of CF intake on exercise recovery: Performance on TT2

Time to complete TT2 was not different between CF intake and PL intake (PL: 30′ ± 1′35″; CF: 29′34″ ± 49″; *p* = 0.41). In both the PL and CF trial, relative power output changed significantly during TT2 (PL: *p* = 0.02; CF: *p* < 0.01), with a significant increase in power output during the last 5 min of TT2. Relative power output was not significantly different between CF and PL at any of the time points. During TT2, glucose, lactate, heartrate and RPE increased significantly (main effect of time), but were unaffected by CF intake (no main effect of trial, no interaction effect) (data not shown).

## Discussion

This study aimed to examine the effect of CF intake on: *i)* indirect markers of eNOS-dependent NO production during exercise, *ii)* exercise-induced changes in antioxidant capacity, oxidative stress and inflammation, and *iii)* exercise performance and recovery.

Epicatechin, one of the main components of CFs, is at least partially associated with the observed improvements in NO synthesis and endothelial function [30]. CF intake increased serum epicatechin concentrations reaching its peak concentration (approximately 450 nM) between 100 and 130 min after intake, exactly during the first exercise bout, i.e. TT1. We hypothesized that CF intake, through the subsequent increase in epicatechin, would increase NO production, which could influence TT performance. In this study, NOS-dependent NO production was indirectly estimated by measuring its substrate L-arginine/ADMA and its by-product citrulline [31], because of our inability to directly assess NOS activity in our experimental model. L-arginine, a rate limiting factor for NO production, is converted into NO and citrulline by eNOS and arginine supplementation has been shown to affect the release of NO [32]. As intracellular ADMA and arginine compete for NOS binding, ADMA and arginine levels regulate NO production [33]. In addition, extracellular ADMA and arginine also compete for cell transport (CAT-2) [34]. Thus, the higher the arginine/ADMA-ratio, the more arginine may be expected to be available as a substrate for eNOS to produce NO. Although it was shown in vitro that CF inhibits arginase activity, L-arginine/ADMA was not increased by acute CF intake in our study.

Exercise-induced shear stress is the principal physiological trigger for eNOS activation and NO production, promoting vasodilation and blood flow [35]. Thus, in this population of well-trained cyclists, NO production is already optimized by their regular training [23]. We examined whether acute CF intake could improve their exercise-induced NO production even more. Citrulline increased and L-arginine/ADMA decreased after both TTs, suggesting an exercise-induced increase in eNOS activity and NO production, but we did not observe an improved eNOS activation by CF as there were no differences in L-arginine/ADMA (eNOS substrate) and citrulline (byproduct) after CF intake, neither in rest, nor in response to exercise. However, recent in vitro [17] and in vivo [18, 19] experiments showed that CF intake activates eNOS and improved NO availability. Thus, untrained subjects or a clinical population suffering from reduced NO bioavailability, would probably be more likely to benefit from the CF-induced increase in NO synthesis [22]. Moreover, a very recent pilot study of Taub et al. [36] showed that chronic (3 months) intake of epicatechin-rich chocolate increased VO_2_max and enhanced mitochondrial efficiency in healthy, but untrained people. Further research measuring NO production (e.g. nitrate, nitrite, nitrosospecies, peroxynitrite, eNOS activity), tissue haemodynamics (e.g. Flow Mediated Dilation, Magnetic Resonance Imaging, Doppler) and mitochondrial efficiency is needed to elucidate the exact role of CF on eNOS activation and NO production in combination with exercise.

In this study, time to complete TT1 was not influenced by CF intake, despite the small significant increase in relative power output at the end of TT1. Increases in RPE, lactate, HR and glucose during TT1 were not affected by CF intake. This suggests that acute CF intake has very limited ergogenic effects in well-trained cyclists. Comparing these results directly with similar studies is difficult because of different exercise protocols, as well as different doses, timing, exact composition of the CF extract and the food matrices of CF intake and placebo used. Davison et al. [3] investigated the effects of the acute intake of dark chocolate containing 247 mg CF (of which 97 mg epicatechin) on physiological parameters during 2.5 h cycling at 60% of their VO_2_max. Similar to our study, heartrate, RPE and respiratory exchange ratio (RER) during the exercise protocol were not affected by CF intake. Exercise performance, however, was not measured or reported in this study. Stellingwerff et al. [7] similarly examined the effects of the acute intake of dark chocolate containing 240 mg CF (of which 89 mg epicatechin) on exercise performance, but used a different exercise protocol. Two hours after CF intake, subjects performed 2.5 h of steady state exercise at 45% of VO_2_max, followed by a 15-min TT. The authors did not find any improvements in the 15-min TT performance. This might be explained by the timing of CF intake and the consequent peak of epicatechin in relation to the exercise protocol: the sum of all epicatechin metabolites reached its maximal concentration (approximately 700 nM) during the steady state exercise (after 190 min) and was already decreased by 65% at the start of the subsequent TT.

The second goal of this study was to examine the effects of acute CF intake on exercise-induced changes in antioxidant capacity, oxidative stress and inflammation and its possible implications for exercise recovery. Post-exercise recovery can be optimized by acute intake of antioxidants by scavenging free radicals [9, 12]. Antioxidant capacity refers to the cumulative action of all antioxidants present in the plasma and is modulated by either radical overload or by exogenous antioxidant intake [37]. UA, a strong scavenger of free radicals, contributes to more than 60% of the total plasma antioxidant capacity. However, UA is also upregulated by exercise as a result of an elevated energy-rich purine phosphate catabolism [38]. Therefore, next to assessing the total antioxidant capacity of plasma as TEAC values, UA plasma concentrations were determined and TEAC/UA was assessed [39]. Consistent with previous research which showed that acute exercise induces oxidative stress and antioxidant capacity [8], TT1 and TT2 increased both TEAC/UA and UA plasma concentrations. In line with our hypothesis that CF intake enhances the antioxidant capacity, TEAC/UA and UA were significantly increased by CF intake, before and after exercise. In the study of Davison [3], resting antioxidant capacity was also slightly increased by acute dark chocolate intake. Their exercise protocol (2.5 h cycling at 60% VO_2_max) was longer and less intense than ours, but also increased TEAC values. However, in contrast to our results, they found that dark chocolate intake blunted the exercise-induced rise in antioxidant capacity. In the study of Allgrove [6], a 2-week dark chocolate supplementation tended to increase TEAC at each time point, independently of exercise, which is consistent with our results. MDA, a by-product of lipid peroxidation, is the most frequently studied marker of oxidative stress during exercise [39]. Although TT1 increased MDA plasma concentrations, this temporal change was not affected by CF intake. Davison [3] and Allgrove [6] used F2-isoprostanes as a marker of lipid peroxidation and found that acute and chronic chocolate intake lowered post-exercise plasma free F2-isoprostane levels. In the study of Wiswedel [1], plasma F2-isoprostane concentrations increased after acute CF (187 mg) intake in rest, whereas plasma TEAC and MDA remained unaffected by CF intake. Thus, we cannot rule out that other biomarkers (such as F2-isoprostane) of oxidative stress might have been affected by CF intake in the present study. Although acute CFs intake upregulated the exercise-induced antioxidant capacity, the resultant oxidative stress (lipid peroxidation) was not decreased, suggesting that the small CF-induced increase in antioxidant capacity is not sufficient to counteract the increase in ROS formation during exhaustive exercise.

The TTs induced an increase in inflammatory cytokines IL-1 and IL-6, but not in TNF-α. Although anti-inflammatory properties of CF are well documented by in vitro and ex vivo studies [15], acute intake of CF did not modify the exercise-induced inflammatory response. Similarly, Davison [3] and Allgrove [6] could not find any beneficial effects of acute and chronic dark chocolate intake on exercise-induced inflammatory markers. IL-6, next to its role as pro-inflammatory cytokine, also serves as a myokine produced by skeletal muscle in response to acute exercise to regulate muscle metabolism [40]. Therefore, the inability of acute CF to blunt exercise-induced IL-6 elevations is not detrimental in this context. However, future research should focus on the effect of chronic CF intake on chronic diseases involving inflammation. Gastrointestinal, nervous, and cardiovascular diseases might benefit from chronic anti-inflammatory properties of CF [41], as evidenced by a lowered TNF-α and IL-6 in type 2 diabetes patients following 6 weeks of CF supplementation [42].

Given that CF intake did not reduce time to complete a second TT, exercise-induced inflammation and lipid peroxidation, a biomarker of oxidative stress, we failed to support the hypothesis that CF intake could enhance post-exercise recovery. However, the finding that acute CF intake increased total antioxidant capacity in response to exercise is promising. Future research should focus on its implications on a broader range of biomarkers of oxidative stress, metabolic and vascular parameters in healthy and diseased populations.

## Conclusion

In a population of well-trained cyclists, acute CF intake upregulated exercise-induced total antioxidant capacity, but did not affect lipid peroxidation and exercise-induced inflammation. Acute CF intake did not alter exercise-induced decreases in L-arginine and increases in citrulline (respect. Substrate and byproduct of eNOS-dependent NO production). Acute CF intake did not improve exercise performance and exercise recovery in a population of well-trained cyclists. Further research on the effects of CF on NO bioavailability, oxidative stress and its consequences in an exercise setting is needed to confirm these data.

## Abbreviations

CF: Cocoa flavanol
eNOS: Endothelial nitric oxide synthase
IL: Interleukin
MDA: Malondialdehyde
NO: Nitric Oxide
PL: Placebo
PPO: Peak power output
RM-ANOVA: Repeated measures analysis of variance
ROS: Reactive oxygen species
RPE: Rate of perceived exertion
TEAC: Trolox equivalent antioxidant capacity
TNF-a: Tumor necrosis factor alpha
TT: Time trial
UA: Urid acid
VO_2_ max: Maximal oxygen uptake
